# Host Genetics and Chlamydia Disease: Prediction and Validation of Disease Severity Mechanisms

**DOI:** 10.1371/journal.pone.0033781

**Published:** 2012-03-16

**Authors:** Isao Miyairi, Jesse Ziebarth, Jonathan D. Laxton, Xiaofei Wang, Nico van Rooijen, Robert W. Williams, Lu Lu, Gerald I. Byrne, Yan Cui

**Affiliations:** 1 Department of Microbiology, Immunology & Biochemistry, University of Tennessee Health Science Center, Memphis, Tennessee, United States of America; 2 Department of Pediatrics, University of Tennessee Health Science Center, Memphis, Tennessee, United States of America; 3 Department of Anatomy and Neurobiology, University of Tennessee Health Science Center, Memphis, Tennessee, United States of America; 4 Department of Molecular Cell Biology,Vrije Universiteit Medical Center, Amsterdam, The Netherlands; 5 Jiangsu Key Laboratory of Neuroregeneration, Nantong University, Nantong, China; 6 Division of Infectious Diseases, National Center for Child Health and Development, Tokyo, Japan; Auburn University, United States of America

## Abstract

Genetic mapping studies may provide association between sequence variants and disease susceptibility that can, with further experimental and computational analysis, lead to discovery of causal mechanisms and effective intervention. We have previously demonstrated that polymorphisms in immunity-related GTPases (IRG) confer a significant difference in susceptibility to *Chlamydia psittaci* infection in BXD recombinant mice. Here we combine genetic mapping and network modeling to identify causal pathways underlying this association. We infected a large panel of BXD strains with *C. psittaci* and assessed host genotype, IRG protein polymorphisms, pathogen load, expression of 32 cytokines, inflammatory cell populations, and weight change. Proinflammatory cytokines correlated with each other and were controlled by a novel genetic locus on chromosome 1, but did not affect disease status, as quantified by weight change 6 days after infection In contrast, weight change correlated strongly with levels of inflammatory cell populations and pathogen load that were controlled by an IRG encoding genetic locus (*Ctrq3*) on chromosome 11. These data provided content to generate a predictive model of infection using a Bayesian framework incorporating genotypes, immune system parameters, and weight change as a measure of disease severity. Two predictions derived from the model were tested and confirmed in a second round of experiments. First, strains with the susceptible IRG haplotype lost weight as a function of pathogen load whereas strains with the resistant haplotype were almost completely unaffected over a very wide range of pathogen load. Second, we predicted that macrophage activation by *Ctrq3* would be central in conferring pathogen tolerance. We demonstrated that macrophage depletion in strains with the resistant haplotype led to neutrophil influx and greater weight loss despite a lower pathogen burden. Our results show that genetic mapping and network modeling can be combined to identify causal pathways underlying chlamydial disease susceptibility.

## Introduction

The genus *Chlamydia* comprises a number of species of highly related obligate intracellular prokaryotic pathogens that cause clinical disease in humans ranging from blinding trachoma [1] and sexually transmitted infection by *Chlamydia trachomatis* [2], community acquired pneumonia by *Chlamydia pneumoniae* [3] and life-threatening respiratory and systemic zoonosis by *Chlamydia psittaci* [4]. In a previous study, we determined that a known QTL on chromosome 11 (*Ctrq3*) [5], [6] containing two polymorphic innate immune genes (*Irgm2* and *Irgb10*) in the family of immunity-related GTPases (IRG) were responsible for the innate difference in susceptibility to a systemic infection to *C. psittaci* among the BXD recombinant inbred strains [7]. Each member of this mouse reference strain set inherits a unique and approximately equal fraction of their genomes from two fully inbred progenitors—strain C57BL/6J (B6 or B) and DBA/2J (D2 or D). These two parental strains differ at roughly 5 million sites across the genome. The set of 80 BXD strains is being used for systematic multiscalar genetic studies of host-pathogen interactions [8], [9], [10]. This large set of genetically related strains can provide comparatively high precision mapping, with a resolution of 1–2 Mb in several cases [7], [11]. Characterization of the disease susceptibility differences between the B6 and D2 parental strains revealed significant differences in *C. psittaci* load, inflammatory responses, and cytokine profiles. While the IRGs have been shown to control *Chlamydia* load [6], [7], [12], alternative immunomodulatory functions of these genes have also been reported [13], [14], [15] making it unclear if IRGs influence disease outcome by regulating pathogen load or by influencing other immunomodulatory functions [16].

Recent advances in high-throughput genomic technologies and computational methods allow us to formulate and test genetic network models without explicit data on molecular function. Translating large-scale genomic data into network models with predictive power is a challenging task. The most valid approach is to systematically evaluate the possible hypothetical network models against data and then select the most probable models for experimental validation. The probability of a genetic network model conditioned on the data can be calculated using Bayesian network methods. A Bayesian network is a graphic probabilistic model representing the dependence structure among multiple interacting variables [17], [18], [19]. The probabilistic modelling provides a natural treatment for the stochastic aspects of biological processes and noisy measurements [20]. Bayesian networks can be used to integrate prior knowledge and new data to capture and express causal relationships [21], [22], [23].

We combined forward genetics and Bayesian network analysis to model the biological pathway of how *Ctrq3* or polymorphisms in immunity-related GTPases (IRGs) confer susceptibility and resistance to *Chlamydia* infection in strains of mice with different genetic backgrounds. We then predicted how individual mice would respond to different intervention and validated these predictions. The model predicted that *Ctrq3* confer protection against disease through macrophage activation, which then controls pathogen load and neutrophil influx. The factor with the greatest impact on disease severity, as quantified by weight change in strains infected with *Chlamydia*, was predicted to be neutrophil influx rather than pathogen load. We validated these predictions experimentally. Thus, our work provides an experimentally validated model for an immune-regulatory function of the IRG containing *Ctrq3* locus in contributing to the control of systemic *C. psittaci* infection.

## Results

### Immune responses and disease severity to *Chlamydia psittaci* infection is controlled by two major genetic loci

**Figure 1:**
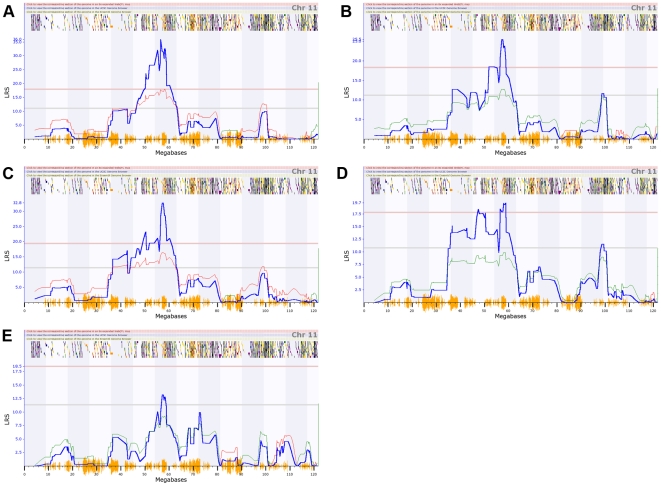
Association of the *Ctrq3* locus with immune parameters and disease status. QTL mapping results for (A) day 6 weight, (B) neutrophils, (C) macrophage activation status, (D) *C. psittaci* load, and (E) G-CSF on chromosome 11. *Ctrq3* is located near 58 Mb on chr 11. Significant (genome-wide adjusted p<0.05) and suggestive (adjusted p<0.63) QTLs are indicated by the solid red and grey lines, respectively. Blue lines indicate the likelihood-ratio statistic (LRS) that the phenotype is associated with the genomic locus. The colored lines following the trend of the LRS show the additive effect of the influence of the locus, with red lines indicating that D alleles increase trait values, while green alleles indicate that B alleles increase trait values.

**Figure 2:**
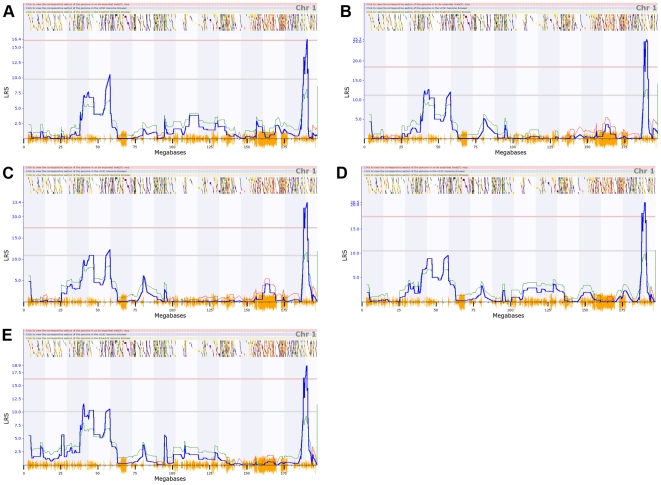
A QTL on chromosome 1 regulating cytokines. QTL mapping results for (A) GM-CSF, (B) IL-1a, (C) MIP-1a, (D) MIP-1b, and (E) MIP-2 on chromosome 1. Significant (genome-wide adjusted p<0.05) and suggestive (adjusted p<0.63) QTLs are indicated by the solid red and grey lines, respectively. Blue lines indicate the likelihood-ratio statistic (LRS) that the phenotype is associated with the genomic locus. The colored lines following the trend of the LRS show the additive effect of the influence of the locus, with red lines indicating that D alleles increase trait values, while green alleles indicate that B alleles increase trait values.

We infected the C57BL/6J parental strain and 40 BXD strains intraperitoneally, and measured peak *C. psittaci* load, levels of macrophages and neutrophils in the peritoneal cavity, 32 cytokines on days 3 and 6; and disease status as quantified by the weight change from the day of infection. Strains exhibited a spectrum of disease ranging from 30% weight loss to 10% weight gain over 6 days. Significant variation in cytokine protein expression was detected for 17 of 32 cytokines (all results will be deposited and will be accessible in GeneNetwork, www.genenetwork.org). We confirmed that the previously mapped and cloned *Ctrq3* locus on chromosome 11 is a major controller of weight change, macrophage activation status (MAS), level of neutrophil recruitment, and *C. psittaci* load on day 6 (Figure 1). A novel secondary locus was mapped to distal Chr 1 at ∼190 Mb. This locus modulates levels of several key cytokines—GM-CSF, IL1a, MIP1a, MIP1b, MIP2—but has no effect on disease severity as measured by weight changes (Figure 2). To further investigate the influence of the genetic polymorphisms at *Ctrq3*, we analyzed the expression pattern of the IRGM2 protein in the peritoneal lavage specimens from infected BXD strains and found that it had two distinct band sizes that are directly correlated with the *Ctrq3* genotype [7].

### Correlation network analysis reveals the immune phenotypes associated with disease severity

**Figure 3:**
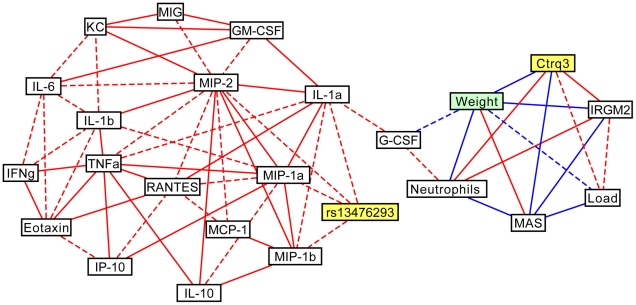
The correlation network of immune parameters during Chlamydia infection in BXD mice. Correlation network linking BXD genotypes (*Ctrq3* and rs13476293), *C. psittaci* load, inflammatory responses, cytokine profiles, IRGM2 protein expression pattern, and weight change after *C. psittaci* infection in BXD strains. Positive (red) and negative (blue) correlations between variables with magnitudes of Pearson's correlation coefficient greater than 0.6 (dashed lines) and 0.7 (solid lines) are shown.

We constructed a correlation network, including cytokines, genotypes, immune parameters and disease phenotypes (Figure 3). The network nodes clustered into two groups. The first group correlated tightly with the *Ctrq3* genotype, IRGM2 expression pattern and several disease-related parameters, including weight change, macrophage activation status (MAS), pathogen load, and neutrophil recruitment. A single cytokine, G-CSF, had a high correlation with weight change and neutrophil level, but was not controlled by *Ctrq3* (Figure 1E. no significant QTL). The second group comprised the cytokines, many of which are highly correlated with each other, and the genotype at rs13476293, a marker located at ∼190 Mb on Chr 1, but not directly with disease-related parameters.

### Bayesian network model identifies the central role of macrophages in the disease pathway

**Figure 4:**
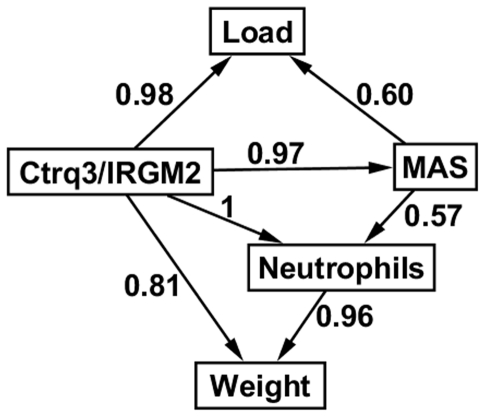
Structure of Bayesian network (BN) model of *C. psittaci* infection. The number next to each directed arc of the BN indicates the confidence (posterior probability) in the arc after model averaging as described in the Methods.

We constructed a Bayesian network model to identify casual pathways through which genotype at *Ctrq3* influences disease outcome after infection with *C. psittaci* (Figure 4). The Bayesian network included variables that were highly correlated with weight change and influenced by the genotype at *Ctrq3*: IRGM2 expression pattern, macrophage activation status, neutrophils, and pathogen load. Because of the perfect correlation between the *Ctrq3* genotype and IRGM2 expression pattern, these variables were combined into a single node. In the most likely model structure, the genotype at *Ctrq3* was the immediate parent of all of the other variables in the model, signifying that each of these variables is directly influenced by the genotype. However, the model also suggests that *Ctrq3* genotype was not sufficient in explaining these variables, as there were additional conditional dependencies in the model structure. Weight change, for example, was directly influenced by neutrophil recruitment and macrophage activation status (MAS) in addition to genotype at *Ctrq3*, indicating that the levels of neutrophils and MAS influences weight change independent of the *Ctrq3* genotype. The model also suggested that macrophage activation influences weight change via regulation of neutrophil recruitment but not by pathogen load restriction.

### Macrophage and neutrophil influx levels defines disease severity

**Figure 5:**
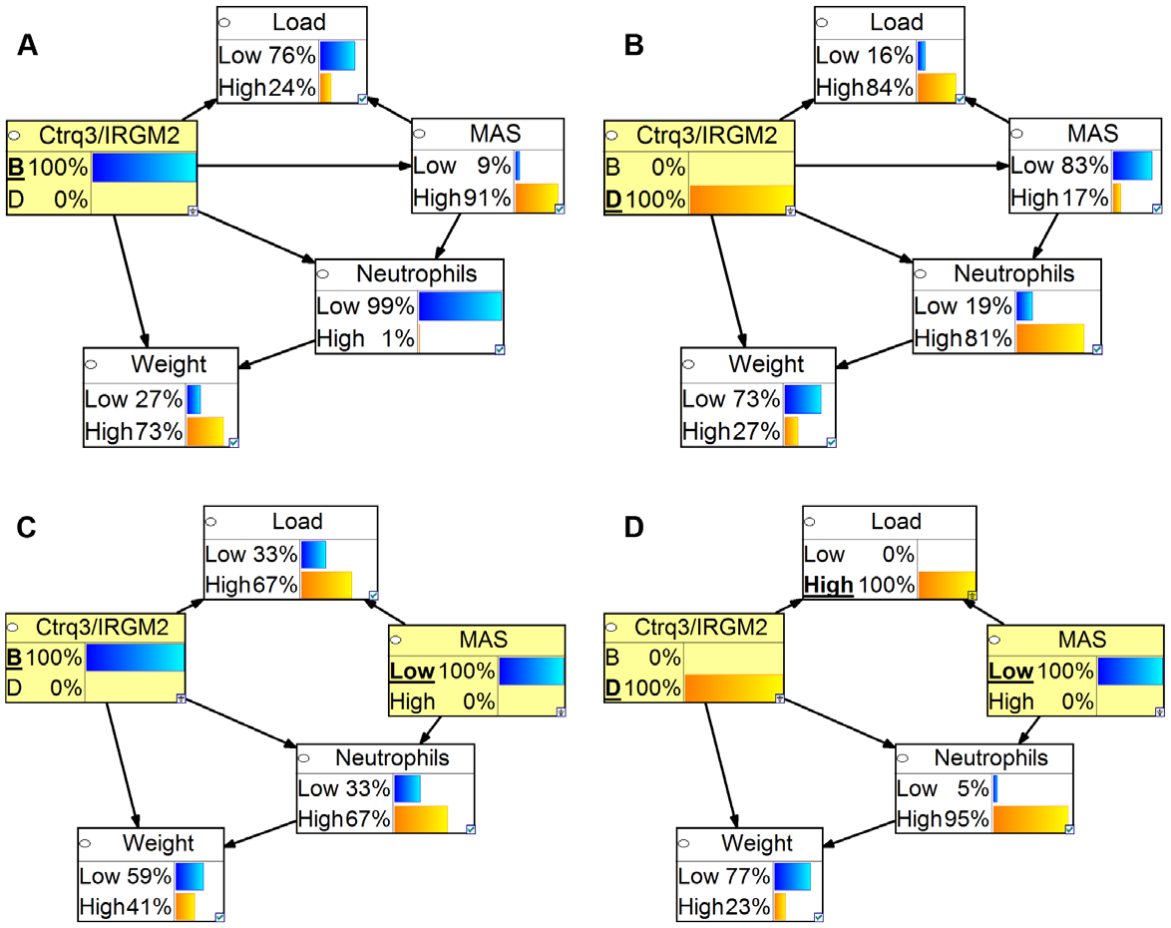
Predictions of the BN as a function of genotype and macrophage intervention. (A and B) Discretized p of the BN as a function of genotype at *Ctrq3*. BXD strains with the susceptible, D, genotype at the IRG locus tend to have lower MAS and weights and higher levels of neutrophils and pathogen load. (C and D) Discretized p effect of interventional depletion of macrophages on the values of variables in the BN.

We used the Bayesian network model to investigate the effect of depletion of macrophages on neutrophil influx, *C. psittaci* load, and weight change (Figure 5 network C and D). To discretize the predictions of the model, a threshold was determined by averaging the mean value of strains with a B genotype and the mean value of strains with a D genotype for the original data sample. Then, the probability that the predicted value for each variable was greater than (High) or less than (Low) this threshold was calculated from the conditional Gaussian distributions learned from the network for the original data and after macrophage depletion (Methods). Nodes with a yellow background have been assigned the value to represent data for only a given genotype and status of intervention on MAS. The magnitude of these changes is expected to be much more pronounced in strains with a B genotype at *Ctrq3* than in strains with a D genotype at this locus. (Figure 5 and Figure S2) Strains with the D genotype at *Ctrq3* typically have innately low macrophage activation, and as a result the model predicts only slight changes in the levels of neutrophils, pathogen load, and weight after depleting macrophages.

**Figure 6:**
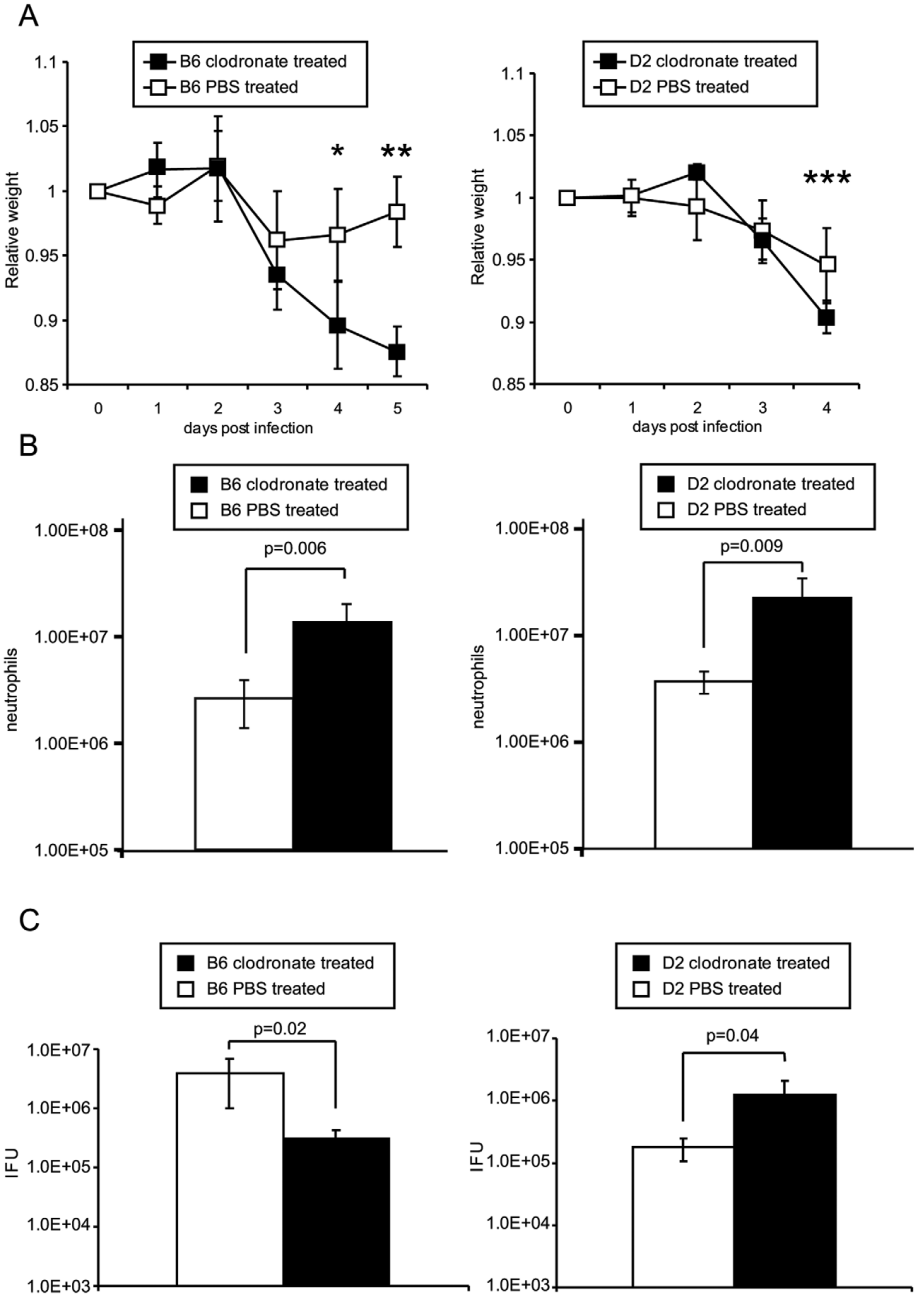
Impact of macrophage depletion on the course of *C. psittaci* infection in B6 and D2 strains. B6 and D2 mice received either liposome clodronate or liposome PBS iv (day −1 post infection) and i.p. (day −1, 1, and 3 post infection) and infected with *C. psittaci* 10^4^ IFU intraperitoneally (N = 5/group). Mice were monitored daily for weight (A) and appearance. Data points where differences in weight met statistical significance are indicated in asterix (* p = 0.003, ** p = 3.7×10E-5, p = 0.04). Clodronate treated D2 strain became moribund on day 4 and B6 on day 5 post infection and were euthanized. Brackets and p values are provided to indicate differences in, number of neutrophils (B), and *C. psittaci* load (C) between clodronate (black) and liposome (white) treated mice for both B6 and D2 strains. Data is representative of two experiments.

We tested these predictions by performing chemical depletion of macrophages with clodronate before infecting B6 and D2 strains with *C. psittaci*. The experiments validated many of the model's predictions (Figure 6). In the D2 strain, depletion of macrophages increased neutrophil influx, *C. psittaci* load, and induced a more rapid decline in weight and mice were therefore euthanized on day 4 post infection. Pathogen load in the liver was greater in macrophage depleted mice by nearly 2 logs (PBS control: 1.21×10^5^ IFU/gram, Clodronate treated; 9.37×10^6^ IFU/gram, p = 0.03). As predicted, in the resistant B6 strain, depletion of macrophages increased neutrophils and exacerbated the weight loss. These mice were moribund 5 days post-infection. Pathogen load in the liver was similar in B6 irrespective of whether macrophages were depleted or not (PBS control: 4.26×10^6^ IFU/gram, Clodronate treated; 3.81×10^6^ IFU/gram, p = 0.06) but their peritoneal pathogen load was decreased after depleting macrophages, suggesting that pathogen load restriction may not be entirely responsible for controlling disease severity.

### *Ctrq3* confers genetic resistance and tolerance to Chlamydia

**Figure 7:**
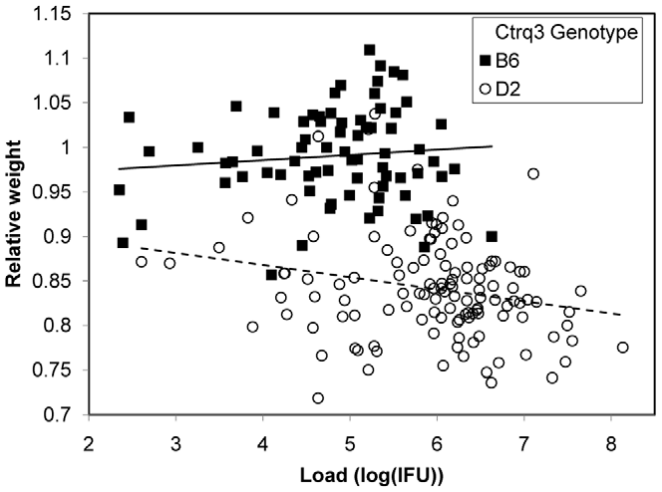
Genetic resistance and tolerance to Chlamydia infection in mice. Plot of weight change as a function of IFU for 197 BXD mice infected with *C. psittaci*. Mice with the susceptible D genotype at the *Ctrq3* (open symbols) lose weight as pathogen load increases, while mice with the resistant B genotype at the *Ctrq3* marker (filled circles) do not. The slopes of the linear regression lines for the B (solid line) and D (dashed line) data are significantly different (p = 0.02).

Several reports have documented that IRGs reduce pathogen burden in vitro and in vivo, which is expected to influence disease severity [6], [7], [12], [24]. To investigate the possibility that the status of the *Ctrq3* genotype switches disease modality, we performed the Bayesian analysis for strains with the B genotype at the *Ctrq3* locus separately from strains with the D genotype. The influence of load on weight change was much stronger for strains with the susceptible D genotype than strains with the resistant B genotype (described further in Methods). Because of the genotype-specific switching between pathogen load and weight change in the model, we expanded this analysis to 197 BXD mice and correlated the weight loss with pathogen load in individual mice according to the genotype at the *Ctrq3* locus (Figure 7). Overall, mice with the B genotype had lower pathogen load compared to mice with a D genotype, although a considerable overlap existed. The mice with the B genotype were tolerant of increases in pathogen burden whereas mice with D genotype lost more weight with increases in pathogen burden as demonstrated by the differences in the slope of the load to weight linear regression lines (*p* = 0.02).

## Discussion

Individualized medicine requires the capability of predicting an individual's susceptibility to diseases and response to medical treatments, based on genetic profile. Individual differences in disease susceptibility and response to therapeutic interventions are complex phenotypes modulated by genetic factors. We formulated an approach using Bayesian networks to model the pathways through which gene variants operate on phenotypes. Results of our study demonstrate experimental validation of the combined systems genetics and Bayesian network approaches to immune pathway modeling and disease prediction. This approach provides a way to develop models for designing genetic association studies that can define causal pathways with the predictive power required in individualized medicine.

We had previously demonstrated that *Ctrq3* controls systemic *C. psittaci* disease outcome and found an association with IRG polymorphisms. While IRGs have cell autonomous functions of pathogen restriction, the immunological pathway that links this genotype to phenotype has not been defined. It has recently been reported that infected hosts employ two different strategies to defend themselves against pathogens—resistance and tolerance [25],[26],[27]. Resistance is defined as the ability to limit pathogen burden, whereas tolerance is defined as the ability to limit the damage caused by a given pathogen burden [28], [29]. While most studies on genetic susceptibility to infectious diseases implicate resistance as a mechanism of host protection, there are several examples of genetic tolerance to infection in animal models [29], [30], [31].

In this study, we predicted that *Ctrq3* conferred resistance but with relatively little impact on weight change. We also predicted an expanded role for *Ctrq3* that included macrophage activation and weight change. Indeed mice with a B6 genotype at the *Ctrq3* locus tended to have a lower pathogen load than mice with D2 genotype and thus were more resistant. However, there was significant variability in the pathogen load within mice with the same genotype suggesting the presence of other factors that affect resistance. The mechanism of resistance by *Ctrq3* is likely to be due to the cell autonomous bactericidal functions of the B6 derived *Irgb10* and *Irgm2* genes given results of our previous ex vivo siRNA experiments [5], [6], [7]. On the other hand, we found that mice with a B6 genotype at the *Ctrq3* locus can maintain body weight over a wide range of pathogen load and thus have tolerance to *C. psittaci* infection. In contrast, mice with a D2 genotype lost weight as a function of increased pathogen burden and were thus less tolerant. The molecular basis of tolerance is still unclear.

An obvious limitation of our model is that we are assessing the function of the 2 MB *Ctrq3* locus. This locus encodes three IRGs (*Irgb10*, *Irgm2*, and *Irgm3*), 18 other genes, as well as non-coding regions with unknown functions. While IRGs remain a primary candidate given its association with immunoregulatory functions [13], [14], [15], [16], [32], [33], it is possible that resistance and tolerance is conveyed by one or more of the other genes in this interval. Furthermore, the exact nature of the B6 and D2 Irgm2 alleles (e.g. “wildtype,” loss-of-function, hypomorph, constitutively active, etc) is unclear and warrant additional biological validation.

While the mechanism of tolerance is unclear, our model suggests that mice that do not recruit activated macrophages to the site of infection have an increased number of recruited neutrophils and more severe disease as evidenced by greater weight loss. Specifically, Bayesian analysis predicted that mice with a D genotype at the *Ctrq3* locus would lose less weight if neutrophils were depleted (day 6 to day 0 weight ratio: 0.83 for mice without neutrophil depletion and 0.89 with neutrophils depleted) without any change in pathogen load. This prediction was consistent with our previous observations where *Cxcr2* knockout mice that cannot recruit neutrophils to the site of infection, survived challenge without any detectible changes in pathogen load. In contrast, the BALB/c wild type strain succumbed to infection with significant neutrophil recruitment in a manner similar to the D2 strain [7]. We speculate that in our model, loss of tolerance leads to uncontrolled inflammation and severe disease high-lighted by neutrophil influx.

Interestingly, we found that macrophage depleted B6 mice have a reduced number of *C. psittaci* in the infected peritoneal cavity; whereas macrophage depleted D2 mice had a greater number. We also found that after macrophage depletion, *C. psittaci* load in the liver of D2 mice increased by 2 logs whereas loads were similar in the liver of B6 mice. We speculate that in B6 mice, loss of a growth niche led to a decrease in pathogen load, whereas the apparent increase in pathogen load in the peritoneal cavity in D2 mice is being supported by an increase in *C. psittaci* growth in the surrounding tissues. While this indicates there may be a difference in tissue/cell tropism between B6 and D2 mice, the underlying mechanism is unknown at this point.

There are clear limitations of our model and approach. First, we are limited by the variables we chose to screen, which did not account for various other cell types, cytokines, physiological parameters, etc. Second, we are limited by the dynamic process of infectious diseases, which include the important variable of time, where our longitudinal analyses were limited (<1 week) due to the severity of disease in D2 mice. Third, we are limited by the nature of the intervention we can employ. In our model, we found that macrophage activation, which occurs gradually over the course of infection, was an important variable that determines disease outcome. In our validation experiment, we eliminated macrophages prior to infection in order to simulate the extreme end of this variable, which may have led to activation of alternative pathways or immune cells. Despite these limitations, our results demonstrate a proof of principal model of how genetic mapping and network modeling can be combined to identify causal pathways underlying infectious disease susceptibility.

## Materials and Methods

### Ethics Statement

This study was carried out in strict accordance with the recommendations in the Guide for the Care and Use of Laboratory Animals of the National Institutes of Health. The protocol (internal protocol number 1709R1) was approved by the Animal Care and Use Committee of the University of Tennessee Health Science Center (PHS assurance# - A-3325-01). No surgical procedures were performed. All efforts were made to minimize suffering.

### Infection and sample collection

*Chlamydia psittaci* infection: *C. psittaci* 6BC was propagated in L cells, titrated and stored at −80C°. Intraperitoneal infection with *C. psittaci* 6BC (10^4^ IFU) was performed using the same stock source to minimize variations across experiments. 8–16 week old male mice (C57BL/6J, and 40 BXD strains) were infected in groups of 2 mice/strain. Infected mice were monitored daily for weight changes. On days 3 or 6-post infection, mice were euthanized to obtain peritoneal lavage samples for pathogen load, flow cytometry, and cytokine analysis. Additional mice, totaling 197 mice representing 56 BXD strains, were infected with *C. psittaci* 6BC (10^4^ IFU) and monitored for weight changes and euthanized on day 6 for IFU analysis.

### Assessment of immune phenotypes

#### *Chlamydia psittaci* load

Titration was performed by a cell culture based IFU assay for day 6 samples as previously described [7]. DNA was extracted from 1 ml of peritoneal lavage fluid from day 3 and *C. psittaci* load was measured as a ratio of *C. psittaci* ompA DNA/host GAPDH by quantitative DNA PCR.

#### Flow cytometry

Standard methods were used as described previously [7]. Briefly, murine peritoneal exudates were blocked with Fc block and incubated with fluorochrome-conjugated antibodies. The following antibodies were used: Macrophage marker; F4/80-APC, Neutrophil marker; Ly6G (clone IA8)-PE, and MHC class II marker; IA/IE-PE. Data was expressed as percent of macrophages or neutrophils in the entire population. MHC class II expression was used as a marker for macrophage activation status and data was expressed as percent of F4/80 positive cells that were also positive for IA/IE.

#### Cytokine analysis

Peritoneal lavage supernatants were analyzed using the Luminex based Mouse 32-plex kit to analyze levels of 32 cytokines (CATALOG).

#### Western blot analysis

Peritoneal lavage specimens were analyzed by Western blot analysis using standard methods with GTPI antibody (M-14) Santa Cruz (sc-11088) and secondary antibody using Goat true blot (eBioscience 18-8814-31).

### Data analysis

#### QTL mapping

Quantitative trait locus (QTL) mapping was performed for 17 cytokine profiles that exhibited variation across strains; immune responses including levels of neutrophils, macrophages, and macrophage activation status (MAS); and weight change of BXD strain infection with *C. psittaci* with the GeneNetwork (www.genenetwork.org). Single marker regression was performed across the entire mouse chromosome at 3795 markers typed across BXD strains. A likelihood ratio statistic (LRS) was calculated at each marker comparing the hypothesis that the marker is associated with the phenotype with the null hypothesis that there is no association between marker and phenotype. Genome-wide significance was determined by performing 1000 permutations. Two significant (genome-wide p-value<0.05) QTLs were found: one QTL was located near 55 Mb on chromosome 11 and was associated with weight change, MAS, pathogen load, and neutrophil levels (Figure 1), while the other significant QTL was located on chromosome 1 near 190 Mb and was associated with several cytokines (Figure 2).

#### Bayesian network modeling

Structural learning of the Bayesian network was performed using the R package *deal* (http://cran.r-project.org/web/packages/deal/index.html). The network was constructed from data for C57BL/6J and 40 BXD strains using one discrete node, representing the *Ctrq3* genotype and IRGM2 protein expression pattern, and four continuous nodes (neutrophils, *C. psittaci* load, macrophage activation status, and the ratio of the weight of the mice 6 days after infection to the weight before infection), which were modeled with conditional Gaussian distributions. The Bayesian network score [34], which is basically a version of the BDe scoring metric [35] extended to include conditional Gaussian distributions, was calculated in *deal* for all, except those that violated two restrictions. First, potential models in which the genotype node was the child of any other nodes in the network were not considered. This restriction does not require that the genotype node be the parent of the other nodes, as model structures in which continuous nodes were independent of genotype were allowed. Second, the weight node could not be the parent of any other node. The Bayesian score metric inherently handles the problem of over fitting data to complex models [36]. However, selecting a single best network model and ignoring all other models may still lead to over-fitting the data. Model averaging can be used to reduce this risk [37]. An indicator function 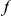 is defined as: if a network G learned from data D has the feature (here a feature is a directed edge representing a regulatory relationship), 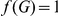, otherwise, 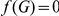. The posterior probability of a feature is 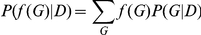. This probability reflects our confidence in the feature 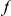. We calculated the posterior probability of features by averaging over all possible models, with the restrictions noted above. All features with a posterior probability greater than 0.5 were included in the network.

The reproducibility of the structure learning method was investigated with the use of simulated data. Briefly, the model learned for the original network was used to generate simulated data sets. Then, the structure learning method was repeated with the simulated data sets and the network structures learned from the simulated data sets were compared to the structure of the original network. A high correspondence between the simulated structures and the original structure indicates that the size of the original data set was sufficient to learn the structure of the network. To create the simulated data, parameter learning of the network was performed with the maximum likelihood estimator provided in the Bayes Net Toolbox [38], available for download at: http://code.google.com/p/bnt/. Before the parameters of the network were learned, the data for the four continuous nodes was normalized to have a mean of 0 and standard deviation of 1. 1000 simulated data sets with 41 samples were then generated with the sample_bnet function of the Bayes Net Toolbox. The structure learning method used to learn the original network was then used for each of the simulated data sets. The edges in the original network were highly reproduced in the simulated data (Figure S1).

#### Prediction of effects of macrophage depletion

We predicted the effects of intervention using a hybrid Bayesian network including both the discrete genotype node and continuous nodes modeled with conditional Gaussian distributions with the Bayes Net Toolbox. The parameters of the network were learned with a maximum likelihood approach. Macrophage depletion is an external intervention to the model. The intervention sets the value of the MAS node and relieves it from the influence of its parent node. Therefore, prediction was performed by removing the link *Ctrq3*/IRGM2→MAS and setting macrophage activation status to the minimum value observed in the data used for parameter learning [action *do* (MAS = MIN), where MIN = 0,032 is the minimum observed MAS value]. The probabilistic inference was executed using the Bayes Net Toolbox. The effects of macrophage depletion on the parameters of the conditional Gaussian distributions for each node are compared in Table S1 and Figure S2. Depletion of macrophages causes increases in the levels of neutrophils and pathogen load and decreased weight. The magnitude of these changes is larger for mice with the D genotype at *Ctrq3*.

#### Bayesian network cross-validation

Leave-one-out cross validation was also used to test the performance of the hybrid Bayesian network. For each test strain, parameter learning of the remaining 40 strains and inference was performed with the Bayes Net Toolbox with the methods mentioned above. To evaluate the quality of the continuous predictions, we used the *Q^2^* parameter [39], which is given by:
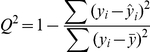
where *y_i_* is the value of the *i*th sample, *ŷ_i_* is the predicted value of the *i*th sample, and 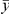 is the sample mean. The values of *Q^2^* for MAS, neutrophil level, pathogen load, and weight were 0.51, 0.59, 0.45, and 0.68, respectively. Additionally, we discretized the original data and the predictions from the leave-one-out-cross validation for each strain and used this discretized data to test the accuracy of the predictions. A threshold for each of the continuous variables was determined by averaging the mean value of the original data for all strains with the B genotype and the mean value for all strains with the D genotype. Then, the continuous variables for the original data and the predictions were classified as being either High or Low through comparison with these threshold values for each strain. The accuracy was then determined by dividing the number of predictions that matched the original data by the total number of strains. For MAS, neutrophil level, pathogen load, and weight, the accuracy was accuracy 85%, 93%, 80%, and 88%, respectively.

Strain dependent influence of pathogen load on weight change after infection: To test if the arc from pathogen load to weight ratio was genotype dependent, the data for mice with B and D genotypes at the *Ctrq3* locus were separated. As each data set only contained data from strains with one genotype, the genotype node was removed from the network, and the structure of the network, using model averaging of an exhaustive search of possible structures with *deal*, was learned for both the B and D data. For strains with the D genotype, a directed edge from pathogen load to weight ratio had a posterior probability of 0.64, while the same edge for strains with the B genotype had a posterior probability of only 0.16, indicating that weight change was dependent on *C. psittaci* load only for strains with the susceptible D genotype. This conclusion was confirmed by grouping the pathogen load and weight ratio for a total of 197 mice into B and D groups using the genotype at *Ctrq3*. Slopes of the linear regression lines of pathogen load vs. weight ratio for each group were calculated and compared using the analysis of covariance tool in Matlab 7.8 (R2009a). The slopes of the lines were significantly different with a p-value of 0.02.

### Validation experiments

*B6* and *D2* mice were each grouped into two groups (N = 5) that received clodronate containing liposome or PBS containing liposome injections on day −1, 1, 3, 5 (day −1: 200 uL iv, 200 uL i.p., day 1, 3, 5: 200 uL i.p.) [40]. All mice were infected on day 0 with *C. psittaci* 6BC at 10^4^ IFU i.p. and monitored daily for weight changes. On day 6 post infection, mice were euthanized and peritoneal lavage was obtained. The peritoneal lavage was processed for pathogen load, cell population (number of neutrophils and macrophages) and macrophage activation status by flow cytometry as described before. The total number of cells in the lavage was enumerated by cytometer and total numbers of neutrophils were calculated.

## Supporting Information

Figure S1 **Reproducibility of network structures.** The number next to each edge is the fraction of times that the edge was present in the structure of 1000 simulated data sets. The simulated data sets were generated from the parameters of the original network and contained 41 samples, the same number of samples as in the original data set. The structure learning method used for the simulated data sets was the same as that used for the original network. No edges not present in the original network occurred in more than 0.29 of the simulated data sets.
(TIF)

Figure S2 **Effect of macrophage depletion on predictions of continuous data by Bayesian network.** Probability density functions for Gaussian distributions describing the predicted values of macrophage activation status (A), pathogen load (B), neutrophils (C), and weight (D) in normal mice (solid lines) and in mice with depleted macrophages after treatment with clodronate (dashed lines). The effect of macrophage depletion has a much larger effect on predictions for mice with the resistant B6 genotype (blue lines) than with the susceptible D2 genotype (orange lines).
(TIF)

Table S1 **Mean values of Bayesian network predictions as a function of genotype and macrophage intervention.**
(DOC)

## References

[1] Burton MJ, Mabey DC (2009). The global burden of trachoma: a review.. PLoS Negl Trop Dis.

[2] Centers for Disease Control and Prevention (2009). Chlamydia Prevalence Monitoring Project Annual Report 2007..

[3] Hammerschlag M, Kohlhoff S, Apfalter P, Mandell G, Bennett J, Dolin R (2009). Chlamydophila (Chlamydia) pneumoniae.. Mandell, Douglan, and Bennett's Principles and Practice of Infectious Diseases. 7th ed.

[4] Beeckman DS, Vanrompay DC (2009). Zoonotic Chlamydophila psittaci infections from a clinical perspective.. Clin Microbiol Infect.

[5] Bernstein-Hanley I, Balsara ZR, Ulmer W, Coers J, Starnbach MN (2006). Genetic analysis of susceptibility to Chlamydia trachomatis in mouse.. Genes Immun.

[6] Bernstein-Hanley I, Coers J, Balsara ZR, Taylor GA, Starnbach MN (2006). The p47 GTPases Igtp and Irgb10 map to the Chlamydia trachomatis susceptibility locus Ctrq-3 and mediate cellular resistance in mice.. Proc Natl Acad Sci U S A.

[7] Miyairi I, Tatireddigari VR, Mahdi OS, Rose LA, Belland RJ (2007). The p47 GTPases Iigp2 and Irgb10 regulate innate immunity and inflammation to murine Chlamydia psittaci infection.. J Immunol.

[8] Boon AC, deBeauchamp J, Hollmann A, Luke J, Kotb M (2009). Host genetic variation affects resistance to infection with a highly pathogenic H5N1 influenza A virus in mice.. J Virol.

[9] Abdeltawab NF, Aziz RK, Kansal R, Rowe SL, Su Y (2008). An unbiased systems genetics approach to mapping genetic loci modulating susceptibility to severe streptococcal sepsis.. PLoS Pathog.

[10] Kotb M, Fathey N, Aziz R, Rowe S, Williams RW (2008). Unbiased forward genetics and systems biology approaches to understanding how gene-environment interactions work to predict susceptibility and outcomes of infections.. Novartis Found Symp.

[11] Mozhui K, Ciobanu DC, Schikorski T, Wang X, Lu L (2008). Dissection of a QTL hotspot on mouse distal chromosome 1 that modulates neurobehavioral phenotypes and gene expression.. PLoS Genet.

[12] Nelson DE, Virok DP, Wood H, Roshick C, Johnson RM (2005). Chlamydial IFN-gamma immune evasion is linked to host infection tropism.. Proc Natl Acad Sci U S A.

[13] Taylor GA (2007). IRG proteins: key mediators of interferon-regulated host resistance to intracellular pathogens.. Cell Microbiol.

[14] Howard J (2008). The IRG proteins: a function in search of a mechanism.. Immunobiology.

[15] Feng CG, Collazo-Custodio CM, Eckhaus M, Hieny S, Belkaid Y (2004). Mice deficient in LRG-47 display increased susceptibility to mycobacterial infection associated with the induction of lymphopenia.. J Immunol.

[16] Hunn JP, Howard JC (2010). The mouse resistance protein Irgm1 (LRG-47): a regulator or an effector of pathogen defense?. PLoS Pathog.

[17] Pearl J (1988). Probabilistic Reasoning in Intelligent Systems.

[18] Pearl J (2000). Causality: Models, Reasoning, and Inference.

[19] Neapolitan RE (2003). Learning Bayesian Networks.

[20] de Jong H (2002). Modeling and Simulation of Genetic Regulatory Systems: A Literature Review.. Journal of Computational Biology.

[21] Needham CJ, Bradford JR, Bulpitt AJ, Westhead DR (2007). A Primer on Learning in Bayesian Networks for Computational Biology.. PLoS Computational Biology.

[22] Rockman MV (2008). Reverse engineering the genotype-phenotype map with natural genetic variation.. Nature.

[23] Friedman N (2004). Inferring cellular networks using probabilistic graphical models.. Science.

[24] MacMicking JD, Taylor GA, McKinney JD (2003). Immune control of tuberculosis by IFN-gamma-inducible LRG-47.. Science.

[25] Raberg L, Sim D, Read AF (2007). Disentangling genetic variation for resistance and tolerance to infectious diseases in animals.. Science.

[26] Best A, White A, Boots M (2008). Maintenance of host variation in tolerance to pathogens and parasites.. Proc Natl Acad Sci U S A.

[27] Schneider DS, Ayres JS (2008). Two ways to survive infection: what resistance and tolerance can teach us about treating infectious diseases.. Nat Rev Immunol.

[28] Medzhitov R (2009). Damage control in host-pathogen interactions.. Proc Natl Acad Sci U S A.

[29] Seixas E, Gozzelino R, Chora A, Ferreira A, Silva G (2009). Heme oxygenase-1 affords protection against noncerebral forms of severe malaria.. Proc Natl Acad Sci U S A.

[30] Ayres JS, Schneider DS (2008). A signaling protease required for melanization in Drosophila affects resistance and tolerance of infections.. PLoS Biol.

[31] Ayres JS, Schneider DS (2009). The role of anorexia in resistance and tolerance to infections in Drosophila.. PLoS Biol.

[32] Feng CG, Zheng L, Jankovic D, Bafica A, Cannons JL (2008). The immunity-related GTPase Irgm1 promotes the expansion of activated CD4+ T cell populations by preventing interferon-gamma-induced cell death.. Nat Immunol.

[33] Bafica A, Feng CG, Santiago HC, Aliberti J, Cheever A (2007). The IFN-inducible GTPase LRG47 (Irgm1) negatively regulates TLR4-triggered proinflammatory cytokine production and prevents endotoxemia.. J Immunol.

[34] Bottcher S (2001). Learning Bayesian networks with mixed variables.

[35] Heckerman D, Geiger D, Chickerling D (1995). Learning Baysian networks: The combination of knowledge and statistical data.

[36] Pe'er D (2005). Bayesian network analysis of signaling networks: a primer.. Sci STKE.

[37] Hartemink AJ, Gifford DK, Jaakkola TS, Young RA (2001). Using graphical models and genomic expression data to statistically validate models of genetic regulatory networks.. Pac Symp Biocomput.

[38] Murphy KP (2001). The Bayes Net Toolbox for Matlab..

[39] Hawkins DM, Basak SC, Mills D (2003). Assessing model fit by cross-validation.. J Chem Inf Comput Sci.

[40] Van Rooijen N, Sanders A (1994). Liposome mediated depletion of macrophages: mechanism of action, preparation of liposomes and applications.. J Immunol Methods.

