# Supplementary figures and images for: Host Genetics and Chlamydia Disease: Prediction and Validation of Disease Severity Mechanisms
Source: PLoS One. 2012 Mar 16;7(3):e33781. doi: 10.1371/journal.pone.0033781 (PMC3306297; doi:10.1371/journal.pone.0033781)

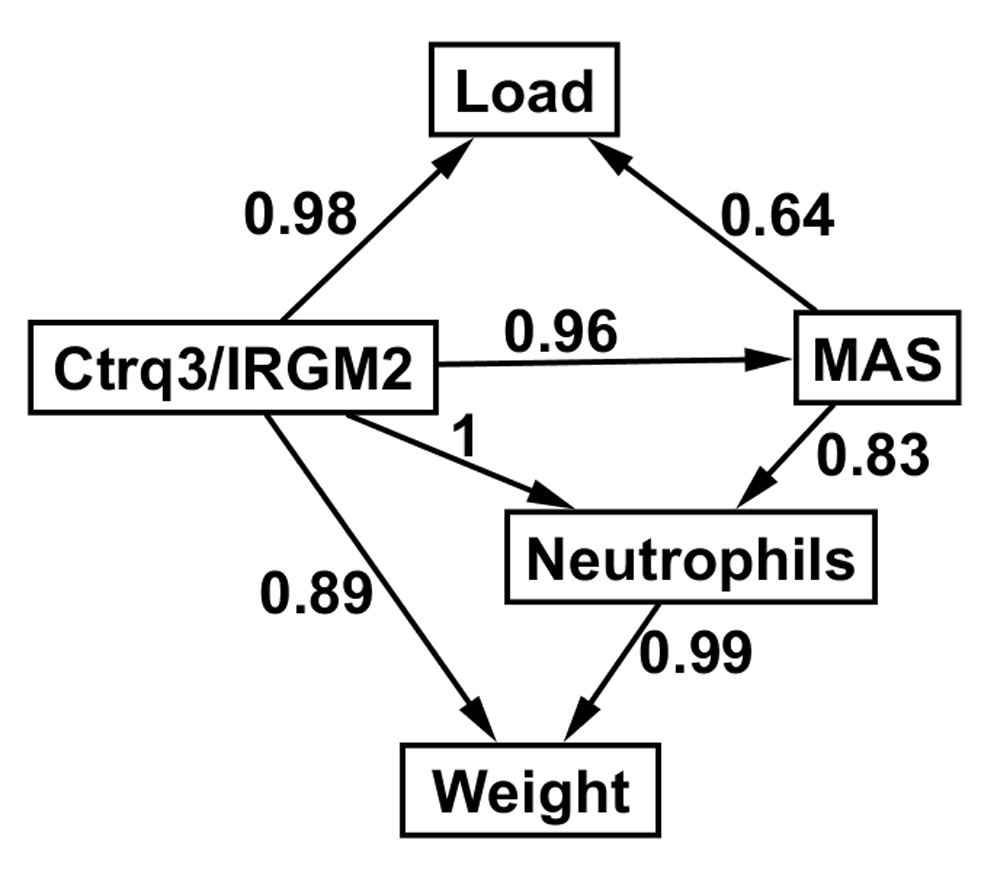

Supplement: Figure S1 — Reproducibility of network structures. The number next to each edge is the fraction of times that the edge was present in the structure of 1000 simulated data sets. The simulated data sets were generated from the parameters of the original network and contained 41 samples, the same number of samples as in the original data set. The structure learning method used for the simulated data sets was the same as that used for the original network. No edges not present in the original network occurred in more than 0.29 of the simulated data sets. (TIF) [file pone.0033781.s001.tif]

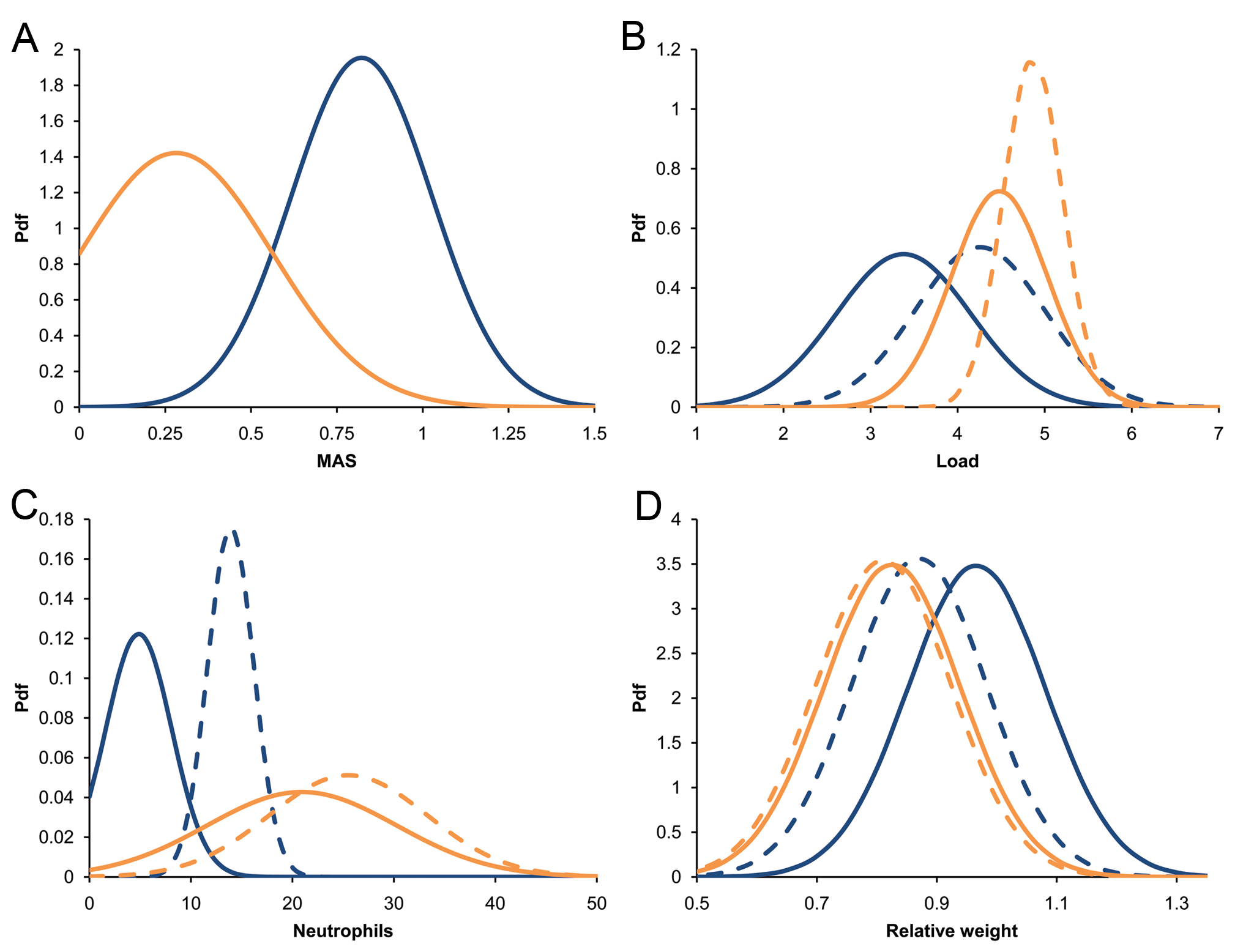

Supplement: Figure S2 — Effect of macrophage depletion on predictions of continuous data by Bayesian network. Probability density functions for Gaussian distributions describing the predicted values of macrophage activation status (A), pathogen load (B), neutrophils (C), and weight (D) in normal mice (solid lines) and in mice with depleted macrophages after treatment with clodronate (dashed lines). The effect of macrophage depletion has a much larger effect on predictions for mice with the resistant B6 genotype (blue lines) than with the susceptible D2 genotype (orange lines). (TIF) [file pone.0033781.s002.tif]
