# Supplementary material for: Host Genetics and Chlamydia Disease: Prediction and Validation of Disease Severity Mechanisms
Source: PLoS One. 2012 Mar 16;7(3):e33781. doi: 10.1371/journal.pone.0033781 (PMC3306297; doi:10.1371/journal.pone.0033781)
Supplement: Table S1 — Mean values of Bayesian network predictions as a function of genotype and macrophage intervention. (DOC) [file pone.0033781.s003.doc]

Table S1. Effect of macrophage depletion on predictions of Bayesian network

|  | B6 original^a^ | B6 MAS depleted^b^ | D2 original^c^ | D2 MAS depleted^d^ |
| --- | --- | --- | --- | --- |
| MAS^e^ | 0.82 | NA | 0.28 | NA |
| Neutrophils | 4.85 | 13.9 | 20.9 | 25.5 |
| Load | 3.4 | 4.3 | 4.5 | 4.9 |
| Weight | 0.97 | 0.87 | 0.83 | 0.81 |

a) Average value of variables for BXD strains with B6 genotype at *Ctrq3*

b) Predicted value of BXD strains with B6 genotype after macrophage depletion

c) Average value of variables for BXD strains with D2 genotype at *Ctrq3*

d) Predicted value of BXD strains with D2 genotype after macrophage depletion

e) MAS: Macrophage activation status
